# Clinical and cognitive insight in schizophrenia and their relationship with neurocognition and metacognition: a narrative review

**DOI:** 10.3389/fpsyt.2026.1931821

**Published:** 2026-09-22

**Authors:** Xiaohui Chen, Yaowu Gao

**Affiliations:** 1 Medical Affairs Department, Henan Provincial Rongkang Hospital, Luoyang, Henan, China; 2 Department of Psychiatry, Pinghu Mental Health Center, Pinghu, Zhejiang, China

**Keywords:** clinical insight, cognitive insight, insight paradox, metacognition, neurocognition, schizophrenia

## Abstract

Impaired insight in schizophrenia is consistently associated with poorer treatment adherence, more frequent hospitalization, and worse functional outcomes, yet the cognitive mechanisms that account for impaired insight remain incompletely specified. The neurocognitive model has long been the dominant explanatory framework but accounts for only a small fraction of the variance in clinical insight. This review synthesizes evidence on clinical and cognitive insight in relation to neurocognition and metacognition, identifies integrative metacognition as the principal explanatory bridge between basic cognitive resources and illness awareness, and develops the translational implications of this organization. Insight is treated as a multidimensional construct that includes clinician-rated awareness of illness and the metacognitive evaluation of one’s own beliefs. Metacognition is shown to encompass distinct construct families with different bearings on insight, of which the integrative tradition shows the most theoretically central and empirically robust associations with clinical insight. Converging but largely indirect evidence is consistent with a multi-factor model in which integrative metacognition is hypothesized to partially mediate the relationship between neurocognition and clinical insight, while cognitive insight functions as an adjacent construct that overlaps with but does not exhaust metacognitive variance. The insight paradox is incorporated as a structural feature of the model, with internalized stigma possibly moderating whether heightened awareness translates into adaptive engagement or into depression and reduced quality of life. Interventions that target metacognitive processes, including Metacognitive Training and Metacognitive Reflection and Insight Therapy, produce measurable improvements in insight outcomes, whereas approaches without explicit metacognitive targets show limited and inconsistent effects. Longitudinal designs, mediation-focused intervention trials, and the integration of emerging candidate mechanisms such as mental time travel are proposed as priorities for consolidating the model. Literature was identified through searches of PubMed, PsycINFO, Embase, Web of Science, and Scopus up to May 2026, focusing on recent peer-reviewed work in adult schizophrenia-spectrum populations.

## 1 Introduction

Schizophrenia is a debilitating disease characterized by insight deficit among its most significant features. Insight deficit is observed in about 50–80% of patients with schizophrenia, and this deficit begins to appear as early as the first episode of schizophrenia and persists throughout the disease (1). Clinical insight encompasses awareness of having a mental illness, the ability to relabel anomalous experiences as pathological, and recognition of the need for treatment (2). A related but distinct construct, termed cognitive insight, captures the capacity to evaluate and revise one’s own beliefs (3). The two facets are only modestly correlated and can dissociate over time (4). Diminished clinical insight is associated with poorer adherence to antipsychotic treatment (5), higher rates of relapse and readmission (6), and worse social and occupational functioning (7). Understanding why insight is so often impaired has therefore become central to efforts to improve outcomes in psychosis.

Explanatory accounts have been dominated by the neurocognitive model, which proposes that deficits in self-monitoring, particularly those linked to executive dysfunction, underlie poor awareness of illness. The empirical yield of this model is modest. Quantitative syntheses consistently show only small correlations between neurocognitive performance and clinical insight (r ≈ 0.17 overall, and at or below r ≈ 0.20 across specific cognitive domains) (8–10), leaving most of the variance in insight unaccounted for. Parallel growth in metacognition research has added further confusion, since the word now refers to at least three traditions in the literature: integrative metacognitive capacity, cognitive biases such as jumping to conclusions, and metamemory monitoring (11). Following the classification used in the metacognition-assessment literature, these three research traditions are treated here as subdivisions of metacognition rather than as separate core constructs. Each tradition relies on different measurement paradigms and may bear differently on insight, yet they are routinely conflated.

Higher insight is also not unambiguously beneficial. Greater awareness of illness has been linked to elevated depression, lowered self-esteem, and poorer subjective quality of life, particularly when internalized stigma is high (12). The present narrative review delineates the four core constructs, examines how neurocognition and metacognition each relate to insight, and articulates an integrative perspective in which metacognition, particularly its integrative narrative form, operates as a bridging mechanism between basic cognitive capacities and clinical awareness of illness. The insight paradox is treated as a feature of this model, and the framework is connected to current metacognitive interventions to clarify why such interventions may improve insight without amplifying its psychological costs. The present review extends prior integrative frameworks (1, 13) by systematically distinguishing among the three metacognitive traditions, incorporating the insight paradox as a structural component rather than an ancillary finding, and linking the model directly to differential intervention targets.

Literature related to the research topic was searched in PubMed, PsycINFO, Embase, Web of Science, and Scopus databases up to May 2026 using keywords including schizophrenia or psychosis, insight, neurocognition, and metacognition along with their subordinate terms. Eligible sources were peer-reviewed, English-language studies of adults with schizophrenia-spectrum disorders. Consistent with the narrative nature of this review, meta-analyses, systematic reviews, longitudinal cohorts, and intervention trials were given priority, and individual cross-sectional studies were included when they addressed constructs or relationships not covered by higher-level syntheses; case reports, conference abstracts, and studies confined to non-psychotic populations were not considered. Bibliographies of important meta-analyses and systematic reviews were hand-searched for identifying more relevant articles.

## 2 Conceptual and measurement foundations

This review is organized around four core constructs: clinical insight, cognitive insight, neurocognition, and metacognition. Clinical and cognitive insight are defined first, followed by neurocognition and then metacognition, which is treated as three research traditions rather than as a single construct: integrative metacognition, cognitive biases, and metacognitive monitoring.

### 2.1 Clinical and cognitive dimensions

Clinical insight means the patient’s awareness of being ill, the ability to reclassify the psychotic phenomena as pathological, and the realization of the necessity for treatment. The three-dimensional model of insight, formulated in 1990, suggested the approach whereby insight was neither a black-and-white concept nor a reflection of total acknowledgment of illness, but rather overlapping and independent dimensions (2). This model is operationalized by using the Schedule for the Assessment of Insight-Expanded version (SAI-E) (14) and the framework is extended by using the Scale to Assess Unawareness of Mental Disorder (SUMD), which covers symptom awareness, symptom attribution to the disorder, and awareness of the social ramifications of the disorder (15). Other assessment measures for self-report include the Birchwood Insight Scale (BIS) (16) and G12 item from the Positive and Negative Syndrome Scale, both of which are widely used but assess insight at different granularities. While scoring procedures differ, both tools agree on the three-factor structure consistent with the original theory, even though there remains heterogeneity in the factor solution and low cross-instrument agreement that hinder comparability (17). The main instruments are compared side-by-side in **Table 1**.

**Table 1:** Insight assessment instruments in schizophrenia.

Instrument	Type	Rater	Format	Dimensions assessed	Scoring direction
SAI-E (14)	Clinical insight	Clinician	11-item semi-structured interview	Awareness of illness; symptom relabeling; treatment compliance	Higher = greater insight
SUMD (15)	Clinical insight	Clinician	Semi-structured interview, general + symptom-specific items	Awareness of mental disorder, effects of medication, social consequences; symptom-specific awareness and attribution	Higher = greater unawareness (poorer insight)
BIS (16)	Clinical insight	Self-report	8 items, Likert format	Illness awareness; symptom relabeling; need for treatment	Higher = greater insight
PANSS item G12 (17)	Clinical insight	Clinician	Single item	Global lack of judgment and insight	Higher = greater lack of insight (poorer insight)
BCIS (3)	Cognitive insight	Self-report	15 items, Likert format	Self-Reflectiveness; Self-Certainty; Composite = SR − SC	Higher composite = greater cognitive insight

Cognitive insight is a metacognitive phenomenon that refers to an individual’s capacity for self-reflection regarding one’s thoughts, being aware of the possibility of being wrong, and correcting distorted views (3). The measure involves the use of the Beck Cognitive Insight Scale (BCIS), which contains 15 questions and is made up of two scales. Self-Reflectiveness assesses willingness to consider alternative possibilities and feedback while Self-Certainty indicates confidence in personal judgment. The calculation of the composite index consists of subtracting Self-Certainty from Self-Reflectiveness, with high values representing increased cognitive insight. The BCIS captures one specific metacognitive process, an evaluative stance toward one’s own beliefs, rather than the broader integrative metacognition or metacognitive monitoring described below, and it was developed specifically within psychosis research.

The insight in the clinical versus cognitive sense has a much more complicated relation than initially expected. While the validity studies have found a strong connection between BCIS Self-Reflectiveness and SUMD-rated insight into mental disorder (3), this correlation is much weaker and more unstable in later studies, particularly among the first episode of psychosis patients (4, 18). In a one year follow up study of the FACE-SZ cohort, BCIS composite scores were found to decrease with time while insight increased on SUMD (19). Among different samples, Self-Reflectiveness is better correlated with insight compared to Self-Certainty which, on the other hand, is better correlated with delusion and neurocognitive functioning (20). The two facets capture overlapping but partially independent processes and should be assessed separately.

### 2.2 Neurocognition

Neurocognitive deficits are among the most consistent attributes of the schizophrenia spectrum disorders and can be more aptly described as the defining feature of the illness, as opposed to an additional result of any symptom or treatment modality (21). The Measurement and Treatment Research to Improve Cognition in Schizophrenia (MATRICS) program has developed a systematized structure of categorizing the cognitive process into seven broad categories: processing speed, attention and vigilance, working memory, verbal learning and memory, visual learning and memory, reasoning and problem-solving, and social cognition (22). This framework is embodied in the ten standardized tests comprising the MATRICS Consensus Cognitive Battery (MCCB), which is now the most commonly used measure in studies involving clinical trials and observation (22). The deficits within these cognitive domains are well known from the time of first presentation onwards and are responsible for much of the impairment that patients experience in daily functioning (21). In terms of self-monitoring, the capacity to detect and evaluate one’s own errors and performance, executive function (including set shifting) has received considerable attention in the insight literature, although empirically its role in clinical insight is small (8).

### 2.3 Metacognition

The term metacognition spans at least three research traditions in the schizophrenia literature, distinguished in the metacognition-assessment literature, each with its own theoretical commitments, measurement procedures, and relationship to insight. Failure to distinguish among them has produced inconsistent findings in the literature (11). The principal characteristics of the three traditions are summarized in **Table 2**.

**Table 2:** Three traditions of metacognition in schizophrenia research.

Feature	Integrative metacognition	Cognitive biases	Metacognitive monitoring
Core construct	Integrated representations of self and others used to address psychosocial challenges	Systematic biases in evidence gathering and belief evaluation	Accuracy of confidence in one’s own cognitive performance
Representative measure	MAS-A, coded from the Indiana Psychiatric Illness Interview narrative (23)	Beads task; Cognitive Biases Questionnaire for Psychosis (24)	Confidence–accuracy calibration; feeling-of-knowing tasks (11)
Level of analysis	Holistic narrative integration	Discrete decision parameters	Trial-by-trial monitoring
Relation to insight	Robust, theoretically central	Growing but heterogeneous	Limited and indirect

The integrative tradition conceptualizes metacognition as the capacity to form increasingly complex and integrated representations of self and others, and to use this understanding to respond to psychosocial challenges (1). For example, a patient who can integrate the experience of hearing voices into an account such as “these are symptoms of my illness that intensify when I am under stress, rather than real external voices” is exercising the narrative integration this tradition describes. Within this model, metacognition is a spectrum of activities including self-reflectivity, awareness of the other’s mind, decentration, and mastery, the application of integrated self-understanding to the demands of daily life. Assessment relies on the Metacognition Assessment Scale-Abbreviated (MAS-A): a personal narrative is first elicited through the Indiana Psychiatric Illness Interview, and the resulting transcript is then rated across these four domains using the MAS-A (23). The tradition is performance-based and narrative in character, and is the conception of metacognition most directly tied to identity and personal recovery (25).

A second tradition treats metacognition as a profile of cognitive or reasoning biases that distort how individuals gather and evaluate evidence. Biases such as jumping to conclusions are reasoning processes rather than metacognitive acts in themselves; they enter the metacognitive literature because noticing, evaluating, and revising such biases is a metacognitive process, and because this line of work has developed under the metacognition label in psychosis research (11, 26). The most studied of these is the jumping to conclusions bias, the tendency to reach decisions on the basis of unusually limited information, classically measured with the beads task; meta-analytic evidence places its association with delusional ideation in psychosis at a small to moderate effect size (27). Related phenomena include overconfidence in errors and a bias against disconfirmatory evidence, both implicated in the formation and maintenance of delusional beliefs (26). The reasoning biases are generally measured via experimental tasks or self-report tools such as the Cognitive Biases Questionnaire for Psychosis (24). The method considers the aspects of metacognition in terms of decision-related factors rather than in terms of story logic.

A third, narrower tradition treats metacognition as monitoring accuracy: the degree to which an individual’s subjective confidence in a cognitive judgment matches its objective correctness. The metamemory perspective is operationalized through confidence-accuracy calibration paradigms, feeling-of-knowing tasks, and judgments of learning (11). Deficits of this kind have been documented in schizophrenia but show less consistent associations with clinical insight than the other two traditions. This approach captures local monitoring at the level of individual judgments rather than the broader self-narrative that clinical insight assessments are designed to elicit, which may account for its weaker connection to awareness of illness.

A complementary phenomenological perspective sits alongside these three traditions and conceptualizes poor insight as an expression of self-disorders (anomalous self-experiences affecting the basic structure of subjectivity rather than reflective or evaluative capacity) that precede psychotic episodes and persist between them (28). This account is conceptually adjacent to the integrative tradition in that both center on the patient’s relationship to self-experience, but locates the disturbance at the level of basic self-awareness rather than at the level of narrative integration. Although the present review focuses on the cognitive-process traditions, the phenomenological account is increasingly cited as a theoretical counterpoint and as a candidate explanation for why some patients with intact neurocognitive and metacognitive performance still show diminished awareness of illness.

### 2.4 Relationships among the four constructs

The four constructs introduced above overlap rather than occupy mutually exclusive territories, and this overlap motivates the integrative perspective developed below. Clinical insight is an illness-related outcome that depends on broader cognitive and metacognitive resources. Neurocognition provides the basic processing scaffolding, including memory, attention, and executive control, on which higher-order reflective activity is built. Integrative metacognition occupies a position between these two layers, drawing on neurocognitive resources to assemble coherent representations of self, others, and lived experience that support the capacity to recognize and accept one’s illness (1). The conceptual relationships among the four constructs are depicted in **Figure 1**.

**Figure 1:**
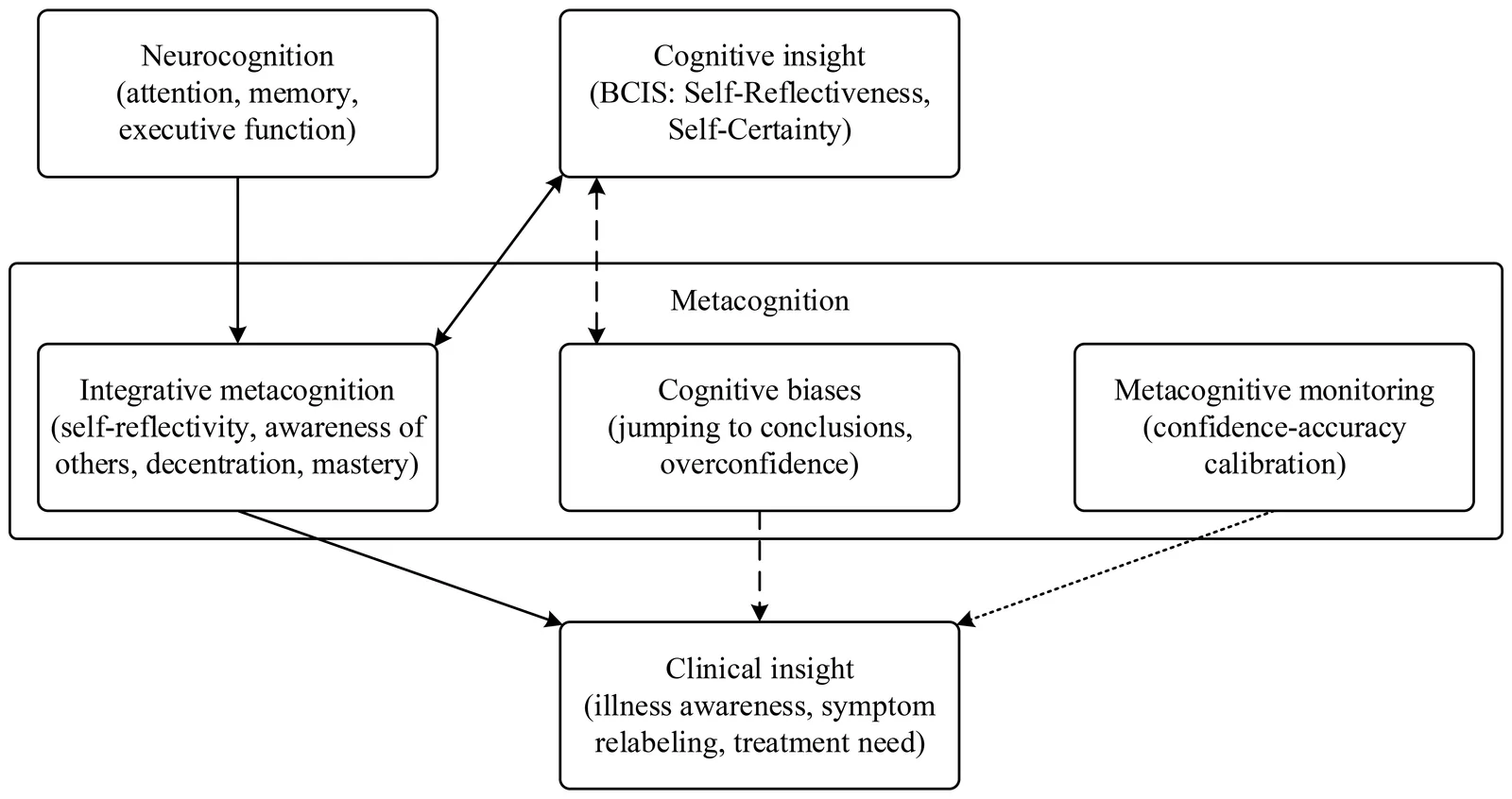
Conceptual map of the four constructs. Boxes denote constructs, and the outer box groups the three metacognitive traditions. Solid arrows denote well-established directional associations (neurocognition supporting integrative metacognition, and integrative metacognition supporting both clinical and cognitive insight); the dashed bidirectional arrow denotes the conceptual overlap between cognitive insight and cognitive biases; the dashed arrow denotes a weaker and more heterogeneous association (cognitive biases to clinical insight); and the dotted arrow denotes a limited and indirect association (metacognitive monitoring to clinical insight).

Cognitive insight sits at the intersection of these two perspectives. The BCIS captures a metacognitive process, namely an evaluative stance toward one’s own beliefs (3), but the construct has historically been classified as a form of insight because the instrument was developed in psychosis research and was intended to address the difficulty patients face in revising delusional beliefs. The BCIS, and especially its Self-Reflectiveness subscale, can therefore serve as a bridge between the cognitive insight tradition and the integrative metacognition tradition. Its Self-Certainty subscale tracks more closely with delusion-related cognitive biases and overconfidence than with the integrative capacity for self-narrative (10, 20). The Self-Reflectiveness–Self-Certainty distinction within the BCIS mirrors the broader division within the metacognitive literature. This alignment is interpretive and does not imply that the two measures are equivalent. The BCIS and the MAS-A are not interchangeable, and the link between Self-Reflectiveness and integrative metacognition rests on indirect evidence that integrative metacognitive capacity mediates the Self-Reflectiveness–clinical insight relationship (29), not on any demonstrated construct identity.

Social cognition, particularly theory of mind, partially overlaps with integrative metacognition but is not interchangeable with it. The MATRICS framework groups social cognition with the other neurocognitive domains (22), whereas the integrative tradition treats it as distinct from the awareness of others’ minds (30). Theory of mind shows closer ties to discrete mentalizing abilities, while integrative metacognition shows closer ties to coherent self–other representations across narrative episodes. The two are empirically correlated but separable: both require representing mental states, yet theory of mind concerns inferring specific mental states in discrete situations, whereas integrative metacognition concerns synthesizing such inferences into an evolving account of self and others; where the two have been examined together they behave as related but non-identical constructs (30).

## 3 Evidence linking insight to neurocognition and metacognition

### 3.1 The neurocognitive evidence line

#### 3.1.1 Theoretical premise

According to the neurocognitive perspective, the recognition of illness entails normal functioning of cognitive processes responsible for assessing internal bodily sensations, comparison of these sensations with past experiences, and updating of representations of self in light of any corrections provided by such assessments. Insight deficits are viewed as the consequences of cognitive deficits rather than the symptoms themselves and can be distinguished from motivational or symptomatic models, which attribute insight problems to denial, demoralization, or symptom content. The frontal-executive system has been the candidate substrate primarily due to its involvement in self-monitoring, error detection and cognitive flexibility, and the model is based on the robust prediction that the extent of the deficit in insight is proportional to the degree of executive dysfunction. The theory is empirically testable as the strong theory predicts high levels of correlation between neuropsychological performance and clinical insight while the weak theory predicts moderate levels of correlation close to the degree of other known neuropsychological correlates of psychosis (9).

#### 3.1.2 Meta-analytic evidence

The neurocognition-insight association has been summarized in three meta-analyses within two decades (8–10), and all of them draw the same general conclusion. The first meta-analysis found that there is a weak correlation between insight and neurocognitive functioning, particularly with the frontal-executive measures (9). A subsequent meta-analysis (10) extended the analysis to specific cognitive domains and reported correlations that did not exceed approximately r = 0.20 for any domain, while introducing a notable dissociation within the BCIS: Self-Reflectiveness was unrelated to neurocognitive performance, whereas Self-Certainty was negatively correlated with memory and IQ. The most recent synthesis, organized around the six MATRICS domains, reported even smaller correlations between neurocognition and insight, with theory of mind reaching a more substantial association than any neurocognitive domain, and symptom domains exceeding both (8). As the pooled studies grew from k = 35 to k = 123 and combined samples reached almost N = 15,000, and as coverage widened from overall and frontal-executive measures to the six MATRICS domains, the magnitude of the neurocognition–insight association remained small and stable. The Self-Reflectiveness–Self-Certainty dissociation within the BCIS points to a more fine-grained picture in which a self-evaluative stance toward one’s own beliefs is unrelated to processing-level capacity, while overconfidence in one’s judgments tracks domain-general cognitive impairment (10).

Neuroimaging evidence broadly parallels these behavioral findings. A meta-analysis of structural and functional brain imaging studies of insight in psychotic disorders identified frontoparietal, temporal, and cerebellar networks (encompassing the precuneus, posterior cingulate, hippocampus, and ventrolateral prefrontal cortex) as the regions most consistently associated with clinical and cognitive insight, without any single region accounting for a large share of the variance (31). The neural correlates of insight are therefore distributed across systems supporting self-reference, memory, and cognitive control rather than localized to executive circuitry, consistent with the modest magnitude observed at the behavioral level.

#### 3.1.3 Evaluation of the neurocognitive line

The magnitude of the neurocognition–insight association is too small to support an explanatory model on its own, and it has not improved across two decades of synthesis. Methodological heterogeneity contributes to but does not erase this gap, since insight measures vary in dimensional structure and scoring method, and different factor structures persistently constrain any direct comparison among studies (17), while first-episode and chronic samples differ in cognitive impairment even though cognitive deterioration in chronic stages adds little relative to pre-existing impairment (32). Because the effect remains small even with improved measures and larger samples, the shortfall appears substantive rather than methodological, so seeking additional layers of explanation is part of the evidence rather than an afterthought.

### 3.2 The metacognitive evidence line

#### 3.2.1 Theoretical premise

According to the metacognitive approach as developed in the integrative metacognition model (1, 13) rather than as a single agreed definition, understanding one’s own disease entails an activity of self-storytelling: it is necessary to integrate strange experiences into a larger story about oneself, how one has become different, and what the meaning of this change is for one’s everyday functioning. Creating an account of this sort necessitates skills that are operationally more complex than mere neurocognitive skills and that are of a different nature from these skills. It entails bringing together fragmented experiences in a self-image that can be evaluated and modified, and making use of this image to address the challenges of daily life. The thesis is not inconsistent with the neurocognitive model, as it comes closer to the insight concept. The neurocognitive line examines the low-level process operations needed for reflective behavior whereas the metacognitive line examines the high-level integration that results in the creation of insight. Both the integrative line and the cognitive bias line have the same underlying assumption, even though they analyze them in different ways (1).

#### 3.2.2 Integrative metacognition and insight

Research studies conducted using MAS-A and its predecessor versions reveal the significant relationship between metacognitive integration skills and insight, with self-reflectivity and mastery being the most related sub-domains (23). Mastery is particularly relevant: the ability to use one’s understanding of mental states to respond to psychological challenges directly enables the formation of a coherent narrative about one’s illness, which is what clinical insight measures are designed to capture. Latent class analysis of a large schizophrenia-spectrum sample further indicates that the good-insight class shows higher MAS-A scores across overall metacognition, self-reflectivity, and mastery than each of the impaired-insight classes, and that the metacognitive advantage persists after statistical adjustment for total symptom severity (33). Group comparisons defined by overlapping clinical and cognitive insight deficits show a similar pattern: co-occurring deficits are accompanied by impaired integrative metacognition without corresponding deficits in executive function or social cognition (34). Across these group-level and latent-class comparisons, metacognitive deficits accompany poor insight whereas neurocognitive deficits do not differentiate insight groups once symptoms are accounted for, a pattern consistent with integrative metacognition contributing to insight beyond basic processing capacity, though this rests on adjusted group contrasts rather than a single effect-size estimate.

#### 3.2.3 Cognitive biases and insight

The cognitive bias approach provides another supplementary perspective on processes involved. This approach identifies the mechanisms involved in the process that allows dysfunctional self-evaluation to perpetuate poor insight beyond just pointing out the existence of dysfunction. The jumping to conclusions bias reduces the threshold of evidence needed for a decision to be made (27). On the other hand, overconfidence in errors and bias against disconfirmatory evidence perpetuate delusional beliefs despite corrective information (26). Both processes map onto the BCIS Self-Certainty subscale, which captures the unwillingness to revise judgments (3). In first-episode psychosis, BCIS Self-Reflectiveness predicts clinical insight more consistently than either Self-Certainty or jumping to conclusions, indicating that the contribution of cognitive biases to clinical insight, while plausible at the process level, is more heterogeneous in empirical observation than the contribution of integrative metacognition (18).

#### 3.2.4 Evaluation of the metacognitive line

Integrative metacognition is more consistently associated with clinical insight than any single neurocognitive domain: it differentiates good- from poor-insight groups where neurocognition does not, even after adjustment for symptoms (33), and cognitive insight predicts clinical insight better than reasoning biases or theory of mind (18). A meta-analysis of MAS-A studies has reported large effects on psychosocial functioning and symptoms (35), but because these outcomes and their effect-size metrics differ from the neurocognition–insight correlations, they are not directly comparable and are cited only as convergent, not equivalent, evidence. The metacognition–insight associations persist after statistical adjustment for neurocognitive performance (34), and converging evidence for a mediation pathway from neurocognition through metacognition to insight is reviewed in the following section. Key meta-analyses and representative empirical studies across both evidence lines are summarized in **Table 3**.

**Table 3:** Studies on insight and its cognitive correlates.

Design	Sample	Insight measure	Predictor	Key effect size	Core finding
Meta-analysis (9)	k = 35	SAI-E, SUMD, BIS	Overall neurocognition; executive function; WCST	r = 0.17 (overall); r = 0.23 (WCST)	Small but reliable insight–neurocognition link; slightly stronger executive component
Meta-analysis (10)	k = 72; N = 5,429 (clinical); k = 7; N = 466 (BCIS)	SAI-E, SUMD, BIS; BCIS	Cognitive domains; BCIS subscales	Clinical: r = 0.13–0.20 across domains; BCIS Self-Reflectiveness n.s.; Self-Certainty r = −0.14 to −0.23	Domain-level neurocognition weakly correlated with clinical insight; BCIS Self-Reflectiveness and Self-Certainty show divergent neurocognitive profiles
Meta-analysis (8)	k = 123; N = 14,932	SAI-E, SUMD, BIS, PANSS G12	MATRICS domains; theory of mind; symptoms	MATRICS r = −0.04 to −0.13; ToM r = −0.23; reality distortion r = 0.28	Largest synthesis to date; neurocognition–insight link remains small even at very large sample
Latent class analysis (33)	N = 324 schizophrenia/schizoaffective	SAI; PANSS	MAS-A; symptoms	Good-insight class higher MAS-A self-reflectivity and mastery than each impaired-insight class	Metacognitive advantage of good-insight class persists after adjustment for total symptoms
Cross-sectional (18)	First-episode psychosis	SAI-E, SUMD	Decision-making; cognitive insight; theory of mind	BCIS Self-Reflectiveness strongest predictor	Cognitive insight predicts clinical insight more consistently than reasoning biases or theory of mind
Group comparison (34)	Schizophrenia-spectrum outpatients	SAI (clinical); BCIS (cognitive)	MAS-A; executive function; social cognition	Group differences on MAS-A only	Co-occurring clinical and cognitive insight deficits accompanied by integrative metacognitive impairment, but not by executive or social cognitive impairment
Mediation analysis (29)	Schizophrenia + early episode psychosis outpatients	SAI	BCIS Self-Reflectiveness; MAS-A	Significant mediation by MAS-A	Integrative metacognition mediates the BCIS Self-Reflectiveness–clinical insight relationship; positive symptoms enter as secondary mediator

## 4 An integrative perspective: metacognition as a bridge

### 4.1 A multi-factor interactive model

The integrative model proposed here brings the two evidence lines together by treating clinical insight as the joint product of basic cognitive resources, higher-order metacognitive integration, symptom-related disruptions, and contextual factors such as internalized stigma. It reorganizes the evidence reviewed above rather than adding new data, and should be read as a proposed structure in which some links are empirically established while the central mediating pathway is advanced as a hypothesis. Integrative metacognition occupies the central position, not because it operates in isolation, but because it is the level at which the contributions of more basic processing capacities, symptoms, and social information are assembled into the self-representation that insight measures attempt to capture (13). This is an interactive as opposed to additive model; this means that there is no single determining factor, but the other factors meet at the metacognitive level and impact the construct through it.

The symptoms can impact the development of insight in two ways within this framework. Reality distortion, disorganization, and negative symptoms are all significantly correlated with low insight (8). This relationship may be partly mediated by metacognition, on the hypothesis that positive or disorganized symptoms disrupt self-narrative and constrain the metacognitive integration on which insight depends (29); because the available data are cross-sectional, a reverse or bidirectional relationship among symptoms, metacognition, and insight cannot be excluded. Social cognition, particularly theory of mind, is related to but distinct from integrative metacognition, insofar as theory of mind focuses on specific mentalization processes whereas integrative metacognition deals with narrative integration (30). Both constructs impact insight via partially dissociated mechanisms even though their impacts tend to be correlated.

Cognitive biases such as jumping to conclusions and overconfidence in errors have a complementary role in the model: They are not alternatives to integrative metacognition; rather, they influence the processes of gathering information that is used by the integrative process (26). The separation between Self-Reflectiveness and Self-Certainty in the BCIS aligns with this dichotomy at the level of measurement, with Self-Reflectiveness relating more closely to the integrative dimension and Self-Certainty to the cognitive-bias dimension; as noted above, this correspondence is interpretive and does not imply that the subscales are equivalent to these metacognitive constructs. Both feed into clinical insight through partially independent pathways.

Internalized stigma enters the model not as a determinant of insight but as a moderator of its downstream consequences. When stigma is high, increased insight is associated with the psychosocial costs documented in the insight paradox literature (36, 37); when stigma is low, the same level of insight tends to support adherence, treatment engagement, and recovery (12, 38). Stigma therefore does not change whether a patient recognizes being ill but changes what that recognition means for the person, which is why interventions that target insight without addressing the stigma context risk producing awareness at the expense of well-being. The relationships among these factors are depicted in **Figure 2**.

**Figure 2:**
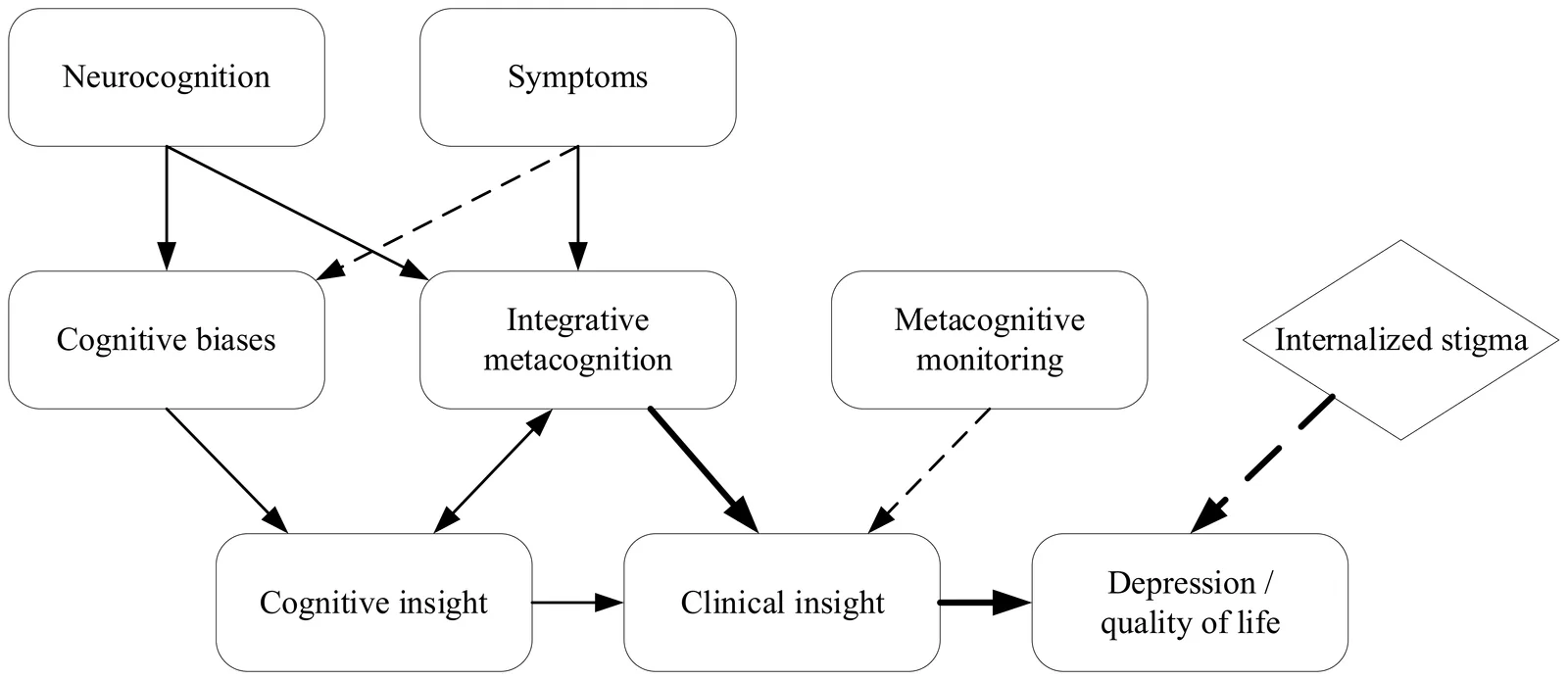
Proposed integrative model of insight. Solid lines denote established associations; bold lines, directly tested mediation or moderation; dashed lines, hypothesized pathways. Clinical insight is the primary outcome; depression and quality of life are downstream consequences.

### 4.2 Mediation pathways: from neurocognition to metacognition to insight

The central hypothesis of the model is that integrative metacognition partially mediates the relationship between neurocognition and clinical insight. Neurocognition supplies the processing resources (attention, working memory, executive control) on which integrative metacognition draws to assemble coherent representations of self and illness, and these representations in turn enable the recognition that one is ill and the relabeling of anomalous experiences as pathological. After this mediated pathway is accounted for, a small direct effect of neurocognition on insight typically remains, so the mediation is partial rather than complete.

Evidence consistent with this mediation is convergent and indirect rather than direct. To our knowledge, no study has yet tested the full proposed pathway from neurocognition through integrative metacognition to clinical insight, so the pathway is best regarded as a hypothesis supported by two adjacent lines of evidence. The first concerns insight as the outcome but does not begin from neurocognition: a direct mediation analysis showed that the relationship between BCIS Self-Reflectiveness and SUMD-rated clinical insight is significantly mediated by overall integrative metacognitive capacity, with positive symptoms entering as a secondary mediator (29). The second begins from neurocognition but concerns functional outcome rather than insight: in first-episode psychosis, metacognition partially mediates the relationship between neurocognition and functional capacity, with neurocognition’s direct contribution shrinking substantially once metacognitive mediation is accounted for (39); a three-year follow-up of the same cohort found that baseline metacognition predicts later functional outcome (40); and a meta-analytic review reported that metacognition relates more strongly to functional outcome than neurocognition does (41). This second line does not test the insight pathway directly, but it supports the broader mediation architecture in which metacognition sits between neurocognition and downstream outcomes. Taken together, the two lines converge on integrative metacognition as an intermediary, while leaving the specific neurocognition-to-clinical-insight pathway to be tested directly.

There are certain shortcomings in the mediation studies. Most studies are cross-sectional in nature, which means that one cannot draw any causal conclusions but has to use the assumption of temporal precedence in order to determine causality. Certain studies have shown partial mediation because the direct effect of the neurocognition-insight link remained statistically significant even when controlled for. Sample sizes for direct mediation analyses on insight are typically modest. The convergence of cross-sectional, longitudinal, and meta-analytic findings, all pointing in the same direction, nevertheless supports the mediation account as the most parsimonious organization of the evidence currently available.

### 4.3 The insight paradox: from antecedents to consequences

The model developed so far addresses what determines whether insight is good or poor. The insight paradox addresses what follows from insight once it is achieved. Three meta-analyses converge on the same finding: greater clinical insight is associated with elevated depression at a small but reliable magnitude (36), with depression mediated by hopelessness and self-esteem and moderated by internalized stigma (42), and with poorer subjective quality of life (37). A similar paradox extends to cognitive insight: BCIS Self-Reflectiveness is positively associated with depressive symptoms, indicating that an evaluative stance toward one’s own beliefs may itself contribute to negative self-appraisal when adaptive psychological resources are absent (43). The same pattern extends to demoralization and reduced hope (1). The relationship to suicide risk is less direct: a systematic review found that insight was only weakly and inconsistently associated with suicide in psychosis, with depression mediating most of the apparent link (44). The more recent discussion revolves around the question of whether “too much insight” can be damaging, although not in an absolute way, where knowledge is bad, but in a conditional one, where knowledge lacking proper psychological coping abilities causes additional problems (45). The concept analysis reveals that the paradox lies in the combination of positive effects (stabilization, treatment compliance, functioning improvement) and negative effects on the subjective level as people gain more insight (46).

The process is reasonably well understood. Internalized stigma appears to moderate the connection between insight and its consequences: where being mentally ill carries personal stigma, becoming aware that one is ill may lead to shame rather than self-management (12). The strength of the moderation effect is dose-related, with the insight-depression and insight-quality of life correlations much greater among populations scoring highly on internalized stigma (37, 42). The integrative metacognitive resources which scaffold the development of insight in the first place, especially mastery (the ability to employ self-knowledge in order to meet challenges adaptively), could conceivably decide if awareness can be converted into healing or not. If mastery exists, insight empowers agency. When there is no mastery, then insight might provide clarity about the self whose actions cannot be taken. These mediation and moderation relationships are drawn from separate samples and meta-analyses and are integrated here conceptually rather than estimated within a single model.

The integration of these results in the model has important implications for designing interventions based on these results. Insight alone is not a valid therapeutic goal because the increase in insight could lead to different results depending on the metacognitive and stigma-related resources of the individual. While an intervention that increases insight but does not address stigma could end up worsening depression and quality of life, an intervention that enhances the ability of integrative metacognition and insight will lead to positive results. The mediation process and the paradox reach the same conclusion that the clinical goal should be integrative metacognition, insight being the result and stigma another issue.

## 5 Clinical implications: metacognition-oriented interventions

### 5.1 Metacognitive training

Where insight depends substantially on metacognition rather than on neurocognitive functioning itself, the most direct interventions target specific metacognitive processes rather than metacognition in general. Two have been most developed: Metacognitive Training (MCT) and Metacognitive Reflection and Insight Therapy (MERIT). Both of these techniques are applicable at two different nodes of the aforementioned integrative model (**Figure 2**), whereby MCT focuses on the cognitive-bias node, whereas MERIT focuses on the integrative-metacognition node. The bases for their evidence vary in development and in their focuses on the consequences that they produce, but both reach the same conclusion regarding the responsiveness of insight to metacognitively oriented interventions.

MCT, first introduced in 2007, represents a group intervention based on cognitive-behavioral approach, yet targeting explicitly biases in reasoning processes which contribute to the emergence of psychotic symptoms (47). This intervention usually includes eight to sixteen weekly sessions in which interactive exercises, visual aids, and discussions help patients become aware of their biases without being accused or challenged. The modules cover jumping to conclusions, excessive confidence in error, attributional biases, theory of mind deficits, and biases in affective cognition. The rationale behind the treatment program is that becoming conscious about these biases and learning different approaches to data collection and interpretation leads to reduced overconfidence in judgments related to delusions and greater openness to alternative explanations measured by the BCIS Self-Reflectiveness sub-scale.

Empirical support for MCT has expanded dramatically within the last twenty years. Positive symptoms and delusions have been demonstrated to decrease consistently in heterogeneous samples (26). Subsequent refinements to the program have included revised modules, content addressing mood and decision confidence, and supplementary tools such as homework exercises and a smartphone application aimed at sustaining effects beyond the treatment period (48). Transfer to basic neurocognitive performance is limited, a finding theoretically coherent given that MCT targets biases at the level of reasoning rather than processing capacity at the level of attention, working memory, or executive control (49).

The effect on insight specifically is the outcome most relevant to the present review. Across trials, the most consistent insight-related finding is a reduction in BCIS Self-Certainty, with smaller and more variable effects on Self-Reflectiveness and on clinician-rated clinical insight measures such as the SAI-E and SUMD (50). The pattern fits the intervention’s mechanism. By directly targeting overconfidence in cognitive judgments, MCT operates at the level the BCIS Self-Certainty subscale was designed to capture, while its impact on broader awareness of illness is less direct and depends on how reduced overconfidence translates into a reorganized understanding of one’s psychotic experiences.

### 5.2 Metacognitive reflection and insight therapy

MERIT was developed as an individual recovery-oriented psychotherapy grounded in the integrative metacognition tradition (51). Unlike MCT, MERIT is open-ended rather than session-limited, individual rather than group-based, and process-driven rather than module-driven. The therapist and patient engage in joint reflection on the patient’s own narrative about themselves, others, and the challenges they face. The aim is not to transmit knowledge about cognitive biases but to support the development of more complex and integrated representations of self and others, and the capacity to apply this understanding adaptively to everyday challenges. The intervention is organized around eight elements that structure therapist behavior without dictating session content, allowing the therapy to track the patient’s evolving metacognitive capacities.

There are procedural and conceptual contrasts between MCT and MERIT. MCT is bottom-up: it corrects discrete reasoning biases so that changes propagate upward into how beliefs are evaluated (47). MERIT is top-down: it strengthens the integrative capacity that organizes self-understanding across episodes of illness, which then reshapes the interpretation of specific experiences (51). The two techniques are not competing but are complementary in their approach to dealing with distinct areas of metacognitive behavior resulting in varied outcomes.

The evidence for MERIT is not as extensive as that for MCT but has certainly grown in recent years. In early psychosis, MERIT achieves significant gains in clinical insight compared to treatment as usual (52). Improvements on the Metacognition Assessment Scale-Abbreviated, particularly on the Self-Reflectivity and Mastery subscales, are also observed at follow-up in a multicenter design (53). The empirical base extends across randomized trials, open trials, case series, and qualitative work, although the flexible and process-based nature of the intervention complicates standardization and dissemination (54). A group adaptation, MERIT-g, is feasible and acceptable in inpatient and outpatient settings, with preliminary effects on recovery-oriented beliefs (55). With respect to insight specifically, the emerging evidence is promising but constrained by modest sample sizes and a limited number of trials with insight as a primary outcome (54).

### 5.3 Metacognition-oriented interventions and insight

A direct quantitative synthesis of metacognitive interventions and insight reports a significant effect on cognitive insight, driven particularly by reductions in BCIS Self-Certainty, while effects on clinical insight are positive but based on fewer studies and more heterogeneous outcome measures (50). The result is consistent with the differential targeting of the two interventions, though no head-to-head trial has compared them: MCT makes up most of the trial population and bears more on cognitive insight, whereas MERIT is the more relevant trial base for clinical insight, a pattern that matches their different targets rather than demonstrating the superiority of either.

Non-metacognitive interventions differ substantially in content, insight measures, populations, and designs, so their results should not be pooled into a single verdict. Psychoeducational techniques, psychoanalytical approaches, cognitive behavioral therapy, self-observation based on video-taped sessions, and pharmacological treatment do not demonstrate any consistent effects on improvement of insight in different types of research (56). A more recent meta-analysis of 21 treatment studies confirmed that the effect of psychoeducation on insight was not significant and the effect of CBT was small, with the most consistent gains observed in studies incorporating metacognitive components (57). For several of these approaches, however, the number of trials and statistical power are limited, so an absence of demonstrated effect should not be equated with a demonstrated absence of effect. A related line of work, therapist-supported computer-assisted cognitive remediation that integrates cognitive training with metacognitive reflection, has improved recovery-oriented outcomes such as self-esteem and meaningful activity in a recent randomized trial, although insight itself was not directly assessed (58).

The convergence is consistent with the primary hypothesis of the empirical theory. If clinical insight requires metacognitive processes to a considerable extent, interventions that involve metacognition would impact insight whereas those that exclude metacognition would have an indirect effect or none at all. The current research body upholds both assumptions. It also conforms to the different mappings suggested in the integrative theory, where MCT acts on the process level layer overlapping with cognitive insight while MERIT acts on the integrative layer overlapping with clinical insight.

The insight paradox poses another implication for the design of an intervention. In addition to enhancing insight, an effective intervention should be able to insulate against the psychological damage caused by heightened insight in the context of internalized stigma. This is something which can be achieved more naturally through one approach rather than through the other. While MERIT enhances mastery and the narrative ability of an individual to incorporate experiences of sickness into their identity (51), it takes a direct stance toward the resources required to offset the impact of the paradox. Whereas MCT mitigates overconfidence and the rigidity of delusion-based reasoning in a relatively indirect way, largely owing to its effect on insight, but not self-narrative (which decides between the demoralizing and adaptive use of any awareness), neither intervention has undergone any investigation within the framework of a study set up to uncover paradox-modulation effects. The combination of the two approaches has been little explored to date.

The available evidence for metacognition-oriented interventions, summarized by intervention type, format, target, outcome, study design, and key finding, is presented in **Table 4**. The pattern across studies indicates that metacognitive interventions can produce measurable improvements in insight, that the effects observed map onto the conceptual targets of each intervention, and that the magnitude and durability of these effects on clinical insight in particular still require larger trials with longer follow-up and explicit examination of mediating mechanisms.

**Table 4:** Metacognition-oriented interventions for insight in schizophrenia spectrum disorders.

Intervention	Format	Metacognitive target	Insight-related outcome	Design	Principal finding
MCT	Group, 8–16 sessions	Cognitive biases (JTC, overconfidence, attributional bias, ToM)	BCIS Self-Reflectiveness, Self-Certainty	Systematic meta-review (26)	Consistent reduction in positive symptoms and delusions across meta-analyses; cognitive insight improvement driven by Self-Certainty reduction
MCT (insight-focused synthesis)	Group, 8–16 sessions	Cognitive biases	Cognitive insight (BCIS); clinical insight (SUMD, SAI)	Systematic review and meta-analysis (50)	Significant effect on cognitive insight across six trials; clinical insight effects smaller and based on fewer, more heterogeneous studies
MCT (neurocognition)	Group	Cognitive biases	Neurocognitive performance (MATRICS-relevant domains)	Systematic review and meta-analysis (49)	Limited transfer to basic neurocognitive performance, consistent with bias-level rather than processing-level targeting
MERIT	Individual, open-ended	Integrative metacognition (MAS-A: self-reflectivity, awareness of others’ minds, decentration, mastery)	Clinical insight (SAI/SUMD); MAS-A subscales	RCT, early-phase psychosis (52)	Significant improvement in clinical insight in MERIT arm relative to treatment as usual
MERIT	Individual, open-ended	Integrative metacognition	MAS-A Self-Reflectivity and Mastery	Multicenter RCT (53)	Improvements on Self-Reflectivity and Mastery at follow-up; effects on secondary symptom outcomes more limited
MERIT-g	Group adaptation	Integrative metacognition	Feasibility, acceptability, recovery-oriented beliefs	Pilot feasibility study (55)	Feasible and acceptable in inpatient and outpatient settings; preliminary effect on recovery-oriented beliefs
Non-metacognitive comparators	Psychoeducation, CBT, psychodynamic, antipsychotics	None explicit	Insight as secondary outcome	Systematic review (56)	Small or non-significant effects on insight; several comparisons underpowered rather than showing a true null

The intervention evidence has notable limitations that constrain the strength of the inferences that can be drawn from it. Sample sizes in the MERIT trial base are modest, follow-up periods in both intervention families are typically short relative to the duration of schizophrenia, and few trials have tested whether the predicted mediator, whether integrative metacognition or cognitive insight, accounts for the observed change in clinical insight. The differential effects of MCT and MERIT on cognitive versus clinical insight remain to be tested in head-to-head designs. The role of stigma as a moderator of intervention outcomes has not been systematically examined, despite its centrality in the paradox literature. These limitations bound the strength of any claim that metacognitive interventions reliably improve clinical insight, and they identify the empirical questions that would most strengthen the evidence base.

## 6 Limitations and future directions

### 6.1 Methodological limitations

There are five limitations inherent to the empirical model which reduce the validity of existing findings and point to the areas for further research. First, the issue of heterogeneity of measurement tools prevents direct comparisons across studies. The main difference lies in the fact that there is dimensionality and scoring diversity among the clinical measures. The factor structure obtained from large multi-national samples does not reliably reproduce itself. BCIS subscales operate in a manner unique to the population being studied (17). Metacognitive assessment tools also vary considerably, as the MAS-A, cognitive bias tests, and metamemory tests each address different concepts and are rarely measured together in a single study (11).

Second, the term “metacognition” continues to refer to at least three distinct construct families in the schizophrenia literature. Failure to specify which tradition is intended has generated findings that appear inconsistent only because they concern different underlying constructs (11). A claim that metacognitive deficits predict insight may involve MAS-A integrative ratings, BCIS subscales, or jumping-to-conclusions performance, with rather different implications for theory and intervention.

Third, the design base remains predominantly cross-sectional. Most evidence linking insight to neurocognition or metacognition consists of correlational observations at a single time point, and although the largest meta-analyses adjust for sample composition and methodological quality, the resulting estimates remain correlational rather than causal (8). Whether metacognitive impairment produces insight deficits, or whether limited insight constrains opportunities for metacognitive development, cannot be settled by this design. Direct mediation evidence with clinical insight as the outcome is limited to a single study and concerns cognitive insight rather than neurocognition as the predictor (29); evidence for the full neurocognition-to-insight pathway remains indirect, and existing analyses rest on modest sample sizes and short follow-up.

Fourth, illness phase is inconsistently controlled. First-episode and chronic samples differ in baseline cognitive impairment, symptom profile, and exposure to antipsychotic medication (32). Although the magnitude of the insight–neurocognition correlation has not diverged systematically across illness phases in the meta-analytic record, the underlying mechanisms may differ, and studies pooling mixed-phase samples may obscure phase-specific patterns.

Fifth, neuroimaging evidence on the neural substrates of insight remains limited. Frontoparietal, temporal, and cerebellar networks are consistently associated with both clinical and cognitive insight, though no region dominates the associated networks (31). The neural correlates of integrative metacognition specifically, particularly its mastery component, remain less well characterized, and the overlap between insight-related and metacognition-related neural networks has not been systematically tested. However, these limitations do not disprove the empirical framework, but rather, place restrictions on the degree to which such an assertion can be made regarding any particular mechanism.

### 6.2 Future directions

Longitudinal and intervention designs are the greatest needs for methodology. The use of longitudinal designs to measure changes in neurocognition, integrative metacognition, cognitive biases, and clinical insight would enable the direct examination of the temporal precedence assumed by the mediation model. Intervention studies involving mediator analyses, which include measurements of the hypothesized mediator as well as insight at several time points, offer the best test possible to confirm whether metacognition mediates the change in insight.

Mental time travel and autobiographical memory represent a promising but still emerging mechanistic hypothesis that has recently started to receive some systematic consideration. One recent narrative review has suggested that impaired episodic future thinking, autobiographical memory specificity, and horizon construction may be involved in the deficits in insight in schizophrenia, via their influence on self-narrative integration and metacognitive monitoring (59). This mechanism connects naturally to the integrative metacognition model, but the evidence is currently limited to a single narrative review and should be treated as a hypothesis rather than an established pathway. The ability to form progressively complex and integrated self-representations depends on the capacity of the individual to mentally revisit specific memories from the past and envision particular future events; as these capabilities become diminished, so does the basis of material on which the process of integrative metacognition constructs its narrative account of the illness experience. Thus, autobiographical mental time travel provides a connection between the cognitive-neuroscientific level of memory and prospection, and the metacognitive-narrative level at which insight is formed, positioning this candidate mechanism above the level of the integrative metacognition node within the model.

It is appropriate to apply network analyses to the multi-component, multi-level architecture of insight research. The modeling of symptoms, neurocognition, metacognition, cognitive insight and clinical insight as nodes, together with the pairwise associations between them as edges, may help uncover central nodes that influence the dynamics of insight-related processes. It may also differentiate them from peripheral nodes and provide possible intervention points which may be undetectable from correlational tables.

The use of digital and ecological momentary assessment techniques provides the possibility of assessing insights and metacognition as they vary in daily life as opposed to assessing these traits as stable variables through clinical testing. Brief probes provided via smartphones may be used to assess symptom awareness, attribution patterns, and metacognitive certainty in nearly real-time fashion, thus allowing a degree of measurement precision not possible with existing questionnaire measures.

Precision intervention represents a translational extension of the mediation model. The differential targeting of MCT and MERIT suggests that patient profiles characterized primarily by cognitive-bias problems (prominent jumping-to-conclusions, elevated BCIS Self-Certainty, modest narrative impairment) may benefit more from MCT, while profiles characterized by integrative-metacognitive impairment (low MAS-A mastery and self-reflectivity, fragmented self-narrative) may benefit more from MERIT (50). Direct empirical tests of patient–intervention matching have not yet been conducted but represent a tractable next step.

Trans-diagnostic extension of the metacognition–insight framework is supported by emerging applications of MERIT and MCT-derived approaches beyond schizophrenia spectrum disorders to bipolar disorder, early psychosis and clinical high-risk states, and personality disorders (54). Whether the relationships among neurocognition, metacognition, insight, and recovery generalize across these conditions, and whether the same intervention targets prove effective, remain open empirical questions of considerable theoretical importance. The methodological priorities identified here do not exhaust the open questions surrounding insight in schizophrenia, but they identify the directions most likely to consolidate or refine the integrative model.

## 7 Conclusion

The phenomenon of insight in schizophrenia is complex and multi-faceted. The connection among clinical insight, cognitive insight and the metacognitive components associated with them cannot be captured within the scope of the neurocognitive theory. Neurocognition provides an essential yet inadequate processing framework. This framework, when supplemented by low correlations between neurocognitive functioning and clinical insight demonstrated consistently for two decades within meta-analysis, marks the explanatory limits of that foundation. Integrative metacognition is placed between basic neurocognitive abilities and clinical awareness of disease, and involves the organization of attention, memory, and executive skills in order to develop the coherent self-representation that is measured by insight measures. The mediation process through which neurocognition affects metacognition, then metacognition affects insight, the different relationship between cognitive and integrative metacognition subdomains and cognitive and clinical insight subdomains respectively, and the structure of the paradox of insight in relation to awareness and internalized stigma together motivate a multivariate model in which metacognition is proposed as the intermediary. This mediation pathway is best read as a hypothesis supported by converging but largely indirect evidence rather than an established causal mechanism. The interventions that rely on metacognition emerge as a direct result of such architecture. Metacognitive Training works at the level of cognitive biases and is more relevant to cognitive insight, while Metacognitive Reflection and Insight Therapy works at the level of integrative metacognition and is more relevant to clinical insight, a correspondence with their respective targets that no head-to-head trial has yet tested. Both intervention programs complement each other instead of competing and both hold great translation potential together by combining insight-based activities along with focusing on internalized stigma because without it all gains made through raising awareness might backfire.

The robustness of these results is limited by the cross-sectional study designs, the diverse range of insight and metacognition tools, and the comparative lack of clinical trials where the mediators of interest have been included along with insight measures. Longitudinal research which follows neurocognition, integrative metacognition, cognitive biases, clinical insight, and stigma over the course of the illness phase would advance this model. Interventional research which puts insight at the center of the research paradigm and includes mediation analysis would also advance this model. The inclusion of additional mechanistic approaches to the model such as mental time travel would further advance this model. Insight in patients with schizophrenia cannot be viewed as an accomplishment, but should be seen rather as a competence that is realized as a result of the maintenance of processing resources, integrative self-representation, and the social significance of being ill. An intervention approach that considers insight in this light is the one that is most justified.
